# Socio-economic status and the double burden of malnutrition in Cambodia between 2000 and 2014: overweight mothers and stunted children

**DOI:** 10.1017/S1368980021000689

**Published:** 2021-05

**Authors:** Michelle K Nakphong, Hiram Beltrán-Sánchez

**Affiliations:** 1 Department of Community Health Sciences, University of California, Los Angeles (UCLA), 650 Charles E. Young Drive South, 36-071 CHS, Box 951772, Los Angeles, CA 90095-1772, USA; 2 California Center for Population Research, UCLA, Los Angeles, USA

**Keywords:** Child stunting, Maternal nutrition, Cambodia, Socio-economic factors

## Abstract

**Objective:**

The Cambodian population has experienced an increase in the proportion of stunted children who have overweight mothers during a period of rapid social and economic growth. We aimed to identify socio-economic factors associated with this household-level double burden over time.

**Design:**

We used data from four Cambodia Demographic and Health Surveys from 2000 to 2014 to study the impact of socio-economic status (SES) on the link between child stunting and overweight mothers in two periods 2000–2005 *v*. 2010–2014. We hypothesised that SES would be a primary factor associated with this phenomenon.

**Participants:**

We included 14 988 children under the age of 5 years, among non-pregnant mothers aged 15–49 years of age and conducted analysis on a subsample of 1572 children with overweight mothers.

**Setting:**

Nationally representative household survey across all regions.

**Results:**

SES factors, specifically household wealth and maternal employment in service or manual occupations (in 2010–2014), are the main drivers of stunting among children of overweight mothers. Children with overweight mothers in the poorest households are more than twice as likely to be stunted than in the richest in both periods (2000–2005: adjusted OR (aOR) = 2·53, 95 % CI: 1·25, 5·13; 2010–2014: aOR = 2·61, 95 % CI: 1·43, 4·77), adjusting for other SES factors, indicating that despite decreasing income inequality, the poorest continue to bear excess risk of a double burden of malnutrition. Maternal short stature also doubled the likelihood of child stunting in both periods, which suggests intergenerational transmission of adversity and physical underdevelopment.

**Conclusions:**

Socio-economic inequalities should be addressed to reduce disparities in the household-level double burden of malnutrition.

Three-quarters of all non-communicable disease (NCD) deaths globally occur in low- and middle-income countries, disproportionately affecting poor and vulnerable populations^(1)^. In order to mitigate the strain of NCD, the WHO has called for efforts to reduce risk factor prevalence^(2)^. Malnutrition at various critical periods of the life course alters metabolic profiles and increases risk for developing NCD^(3,4)^. Of particular concern is a dual burden of malnutrition characterised by concurrent under- and over-nutrition, which has been documented in low- and middle-income countries. This phenomenon may manifest within the same household, such as the occurrence of mothers classified as overweight (‘over-nutrition’) who have children that are stunted (‘under-nutrition’)^(5)^. Seemingly paradoxical, both conditions require unique interventions, yet both also increase the risk of chronic disease onset in later life^(6,7)^.

It is estimated that nearly half of the world’s population affected by under- and over-nutrition reside in southeast Asia and the Pacific^(8)^. This phenomenon has also manifested in Cambodia, where the prevalence of overweight status and obesity has risen remarkably in the last two decades; for example, 6 % of women aged 15–49 in 2000 were classified as overweight or obese compared to 18 % in 2014^(9)^. The rise in overweight status has been associated with the nutritional transition^(10)^, a process by which rapid changes in dietary and physical activity patterns in the population lead to accelerated increases in child and adult overweight/obesity. Yet, survey findings from 2014 in Cambodia indicate that one in three children under the age of 5 years are classified as stunted and about one-fourth (26 %) of stunted children have an overweight or obese mother^(9,11)^. However, little is known about socio-economic factors that link with under- and over-nutrition in Cambodia, despite numerous studies in other countries in southeast Asia^(8,12,13)^.

Because health can be viewed as a general indicator of wealth and social and economic change^(14)^, more knowledge is needed about how socio-economic change relates to health. Evidence indicates that epidemiological and nutritional transitions have been linked to important economic determinants. For example, economic development often corresponds with increased consumption of simple and refined carbohydrates, fat, and processed foods^(15)^. Moreover, health and disease trends exhibit clear social gradients: those who are relatively disadvantaged continue to experience poorer physical health outcomes before and after epidemiological transitions^(16,17)^. This suggests that socio-economic determinants such as wealth, education and social status (e.g. occupation, residence) likely influences health through multiple pathways^(14,18)^.

In addition, an examination of socio-economic factors may provide information about a country’s progression through the nutritional transition. Studies have found contradictory results regarding the link of socio-economic status (SES) with a household double burden of malnutrition (i.e. overweight mothers and stunted children across countries)^(19)^, which indicates that as the nutritional transition unfolds in a population, the SES gradients in the link between maternal weight status and child stunting may change direction. For example, high SES has been associated with overweight mothers with stunted children in South Asia^(20–22)^, a region where the nutritional transition is in the early stages, while low SES is associated with overweight mothers with stunted children in Latin America^(23,24)^, a region that has moved beyond the early stages of the nutritional transition^(10,25)^.

Cambodia has undergone notable social, economic and political change within the past three decades and related factors such as urbanisation, industrialisation and income inequality have been linked to changing health status. Between 1995 and 2018, Cambodia was considered one of the fastest growing economies in the world with an average annual GDP growth of 7·7 %,^(26)^. However, economic growth was also accompanied by income inequality in the mid-2000’s, which rose between 2004 and 2007, and steadily declined after 2007^(27)^. Thus, we expect socio-economic factors to play a differential impact on mothers’ and children’s health status in the early 2000’s relative to recent times when income inequality has receded. In addition, studies conducted in Cambodia during that period have found that urbanisation and industrialised diets were associated with overweight status, as predicted by the nutritional transition^(10,25)^, and that lower SES consistently predicted child stunting^(11,28)^. However, we do not know how SES may impact the occurrence of mothers classified as overweight (‘over-nutrition’) who have children that are stunted (‘under-nutrition’), particularly during the period of rapid economic and industrialisation change in the country. This study addresses these research gaps by assessing the role of SES in shaping the link between overweight mothers and stunted children between 2000–2005 and 2010–2014 in Cambodia, adjusting for maternal health and child characteristics. We sought to understand how these factors reflect broader context-specific population trends and to identify important household-level risk factors for simultaneous over- and under-nutrition^(29)^.

## Methods

### Data

We used data from the Cambodian Demographic and Health Surveys (CDHS)^(9)^, a standardised population-based survey administered in 2000, 2005, 2010 and 2014. Data from the four surveys were appended and pooled into two wave cycles to increase sample size and to capture major economic changes in the country: Period 1 included waves 2000 and 2005 (a period of increasing income inequality) and period 2 included waves 2010 and 2014 (receding income inequality). This design allowed us to compare changes in the role of SES in shaping the link between overweight mothers and stunted children within the decade in which women’s overweight status increased threefold^(9)^. The CDHS questionnaire contains anthropometric data from a subset of children under 5 years of age and their mothers who were aged 15–49 years, who were not pregnant at the time of survey (*n* 15 088 total: *n* 7098 for period 1 and *n* 7990 for period 2). The CDHS also collected socio-economic data about assets, services and living conditions that formed the basis for wealth quintiles, as well as education and occupation^(30)^. We included all mother–child pairs for which anthropometric data were available. In period 1, we excluded the few respondents with missing values in the variables of interest: mother’s BMI (*n* 37), maternal short stature (*n* 2) and maternal employment (*n* 26). The final analytic sample for period 1 comprised 7033 children and 5193 mothers. In period 2, we excluded respondents with missing values in mother’s BMI (*n* 20), maternal short stature (*n* 1), maternal employment (*n* 13) and maternal smoking (*n* 1). The final sample for period 2 comprised 7955 children and 6510 mothers. Of the final sample of children in both periods (*n* 14 988 = 7033 + 7955), 7642 children (51·0 %) had no siblings included in the sample (i.e. unique mothers); 5398 children (36·0 %) had one sibling (i.e. one mother with two children); and the rest, 1948 children (13·0 %), had two or more siblings (i.e. one mother with 3–6 children).

### Child stunting

Stunting was defined as children whose height-for-age Z-score was below minus 2 sds from the median of the WHO child growth standards^(31)^. All children younger than 5 years of age were included in the analysis. A dummy variable was created as follows: a value on 1 was assigned to stunted children and a value of zero to non-stunted.

### Overweight mothers

Overweight status was defined as a BMI equal to or greater than 25·0 kg/m^2(32)^. BMI was calculated by dividing the mother’s weight (in kg) by the square of her height in metres. BMI has been shown to be an adequate measure of obesity and consistently independent of height on both theoretical and empirical grounds^(33)^. A dummy variable was constructed with 1 corresponding to mothers who were overweight.

### Socio-economic and health indicators

We included three categories of covariates based on factors mentioned in related literature: household SES, maternal health and child characteristics. SES included national wealth quintiles, maternal education and maternal employment^(8,34–36)^. Household wealth quintiles, a measure of relative wealth differences, were constructed by the CDHS based on an index of reported assets and household characteristics^(30)^ corresponding to: poorest, poorer, middle, richer and richest. To understand how wealth changed over time, we also examined household assets and access to services – absolute measures of wealth – using a modified EquityTool wealth index comprising nine key assets collected across surveys (Cambodia EquityTool (Released November 2016), equitytool.org, maintained by Metrics for Management). The EquityTool is an abbreviated wealth index that utilises indicators collected in the CDHS surveys with demonstrated high reliability and validity^(37)^. This index included the following indicators: electricity, television, refrigerator, wardrobe, motorcycle/scooter, source of drinking water (wet season), floor material, cooking fuel and toilet facility. Maternal education referred to highest level of educational attainment and was categorised into four groups: no education (completed 0 years of schooling), primary education (completed 1–6 years of schooling), secondary education (completed 7–12 years of schooling) and higher education (completed more than 12 years of schooling). Maternal employment was categorised into three groups: not working, professional/technical/clerical/sales and agricultural/services/manual. Maternal health indicators included maternal age, smoking status and maternal short stature. Smoking status was defined by self-report of current smoker status, including smoking of any substance or use of tobacco in any form. Given that short maternal height is a known predictor of poor child growth^(38)^ and a measure of mothers’ own growth failure (assuming that those aged 18 years and over have reached their full maturity)^(3,36,39,40)^, we included an indicator of maternal short stature. Maternal short stature was indicated by a mother’s height less than 150 cm. Similar definitions of short stature have been used in other health studies^(42–44)^. Within this sample, maternal stature did not predict overweight status (Kruskal’s *λ* = 0·000). Finally, we also included an indicator of whether the mother was born during the Khmer Rouge famine in the period from 1975 to 1979. Birth of mothers during the Khmer Rouge famine has been positively associated with child stunting in Cambodia, suggesting a transgenerational link between intra-uterine exposure to famine, maternal stature and subsequent child stunting^(45)^. Child characteristics included child’s age and sex.

### Analysis

We conducted two sets of analyses to assess SES related to the phenomenon of overweight mothers and stunted children. Specifically, we investigated (1) factors associated with child stunting including maternal overweight status among all mother–child pairs and (2) SES factors associated with child stunting among the subsample of children with overweight mothers. For the analysis of all mother–child pairs, we compared coefficient estimates from a series of logistic regression models in each period that sequentially adjusted for wealth quintile and maternal health indicators and characteristics (mother’s age, parity, education, employment, smoking, short stature and birth during the Khmer Rouge). For the association between child stunting and maternal overweight status in the full sample, we also included child characteristics (child’s age and sex). Diagnostic tests showed approximately linear relationships between continuous variables and the logit of stunting, there were no influential outliers (absolute standardised residuals were all below 3), and variance inflation factors showed there was no multicollinearity between variables. Model fit was assessed and compared by the area under the receiver operating curve. Because our sample included multiple children from the same mother, we used robust standard errors to correct for clustering by mother. Sensitivity analyses tested potential confounding factors (e.g. region, as regions differed by socio-economic development; and children’s age, as stunting varies by age) and possible joint effects between maternal overweight status and socio-economic factors (e.g. via testing statistical interactions between maternal overweight status and wealth, education, and employment status, separately). We compared models with and without the aforementioned variables using Bayesian information criterion. These analyses indicate that stunting did not differ by region, children’s age was an important factor in the full sample of children but not among those of overweight mothers, and that all SES factors appeared to function independently. Analyses were conducted using StataSE 15.1 (StataCorp. 2017. Stata Statistical Software: Release 15).

## Results

**Fig. 1:**
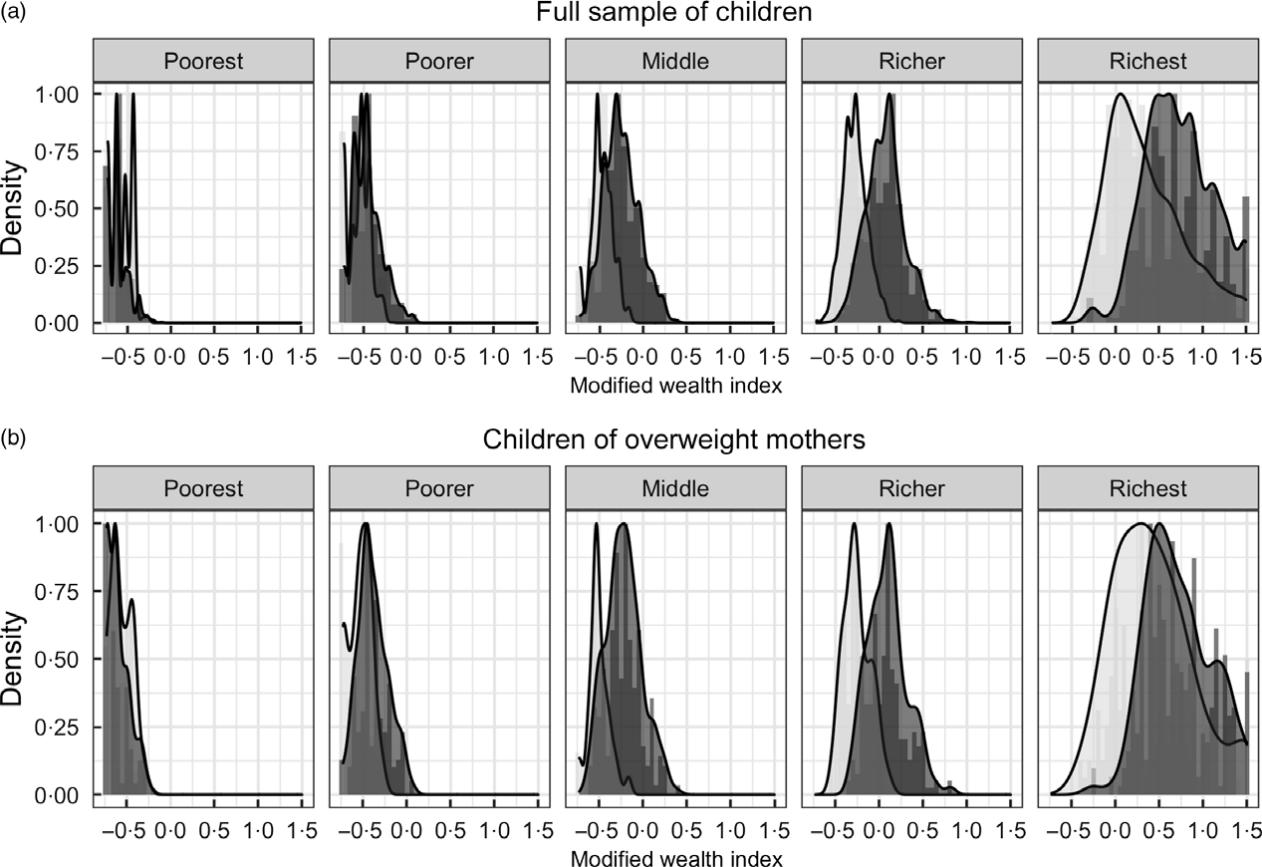
Distribution of wealth indices by wealth quintiles and period among (a) the full sample of children. Period 2000–2005; 2010–2014 and (b) the subsample of children of overweight mothers. 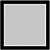, 2000–2005; 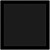, 2010–2014

Descriptive characteristics of the full sample of children in both periods are shown in Table 1. In period 1, 42·4 % of children were stunted while in period 2 this proportion decreased to 30·4 % (*P* < 0·001). In contrast, the proportion of children with overweight mothers increased from 6·5 % in period 1 to 14·0 % in period 2 (*P* < 0·001). Shifts in household wealth over time, as measured by the wealth index, show an increasing wealth gap between the poorest and richest (Fig. 1). Notably, among the poorest quintile, household wealth decreased from period 1 to 2 (*P* < 0·001). Richer wealth quintiles displayed greater household wealth in period 2 as compared to period 1, with greater gains among the richest quintiles.

**Table 1:** Characteristics of the full sample of children by period and stunting status (period 1, *n* 7033; period 2, *n* 7955)

	Period 1 (2000–2005)			Period 2 (2010–2014)		
	Stunted	Not sunted	*P*-value	Stunted	Not stunted	*P*-value
Total number in group	2985	4048		2417	5538	
Maternal weight status						
Not overweight	94·3 %	92·8 %	0·012	89·2 %	84·6 %	< 0·001
Overweight	5·7 %	7·2 %		10·8 %	15·4 %	
Household wealth						
Poorest	33·4 %	23·6 %	< 0·001	32·9 %	20·9 %	< 0·001
Poorer	24·7 %	21·9 %		22·8 %	17·4 %	
Middle	19·7 %	20·1 %		17·3 %	16·1 %	
Richer	14·0 %	16·8 %		14·6 %	18·9 %	
Richest	8·2 %	17·6 %		12·4 %	26·7 %	
Maternal short stature						
Not short	75·3 %	62·7 %	< 0·001	57·8 %	76·2 %	< 0·001
Short	24·7 %	37·3 %		42·2 %	23·9 %	
Mother's highest level of education						
No education	38·9 %	28·5 %	< 0·001	23·2 %	15·2 %	< 0·001
Primary education	52·0 %	54·8 %		54·2 %	49·8 %	
Secondary education	9·1 %	16·4 %		21·6 %	31·5 %	
Higher than secondary education	0·0 %	0·4 %		1·0 %	3·5 %	
Maternal employment						
Not working	32·1 %	35·0 %	< 0·001	29·5 %	35·2 %	< 0·001
Professional/technical/clerical/sales	11·2 %	16·8 %		15·7 %	22·5 %	
Agricultural/services/manual	56·7 %	48·2 %		54·8 %	42·3 %	
Residence						
Urban	15·4 %	19·0 %	< 0·001	19·9 %	29·7 %	< 0·001
Rural	84·6 %	81·0 %		80·1 %	70·3 %	
Maternal age (in years)						
15–24	19·2 %	23·9 %	< 0·001	24·8 %	27·3 %	< 0·001
25–34	48·1 %	48·5 %		54·8 %	56·5 %	
35–49	32·6 %	27·6 %		20·4 %	16·3 %	
Mother's total number of births						
Mean	4·2	3·63	< 0·001	2·96	2·45	< 0·001
sd	2·52	2·29		1·97	1·56	
Maternal smoking status						
Non-smoker	77·2 %	86·4 %	< 0·001	87·6 %	95·0 %	< 0·001
Smoker	22·8 %	13·6 %		12·4 %	5·0 %	
Mother born during Khmer Rouge (1975-1979)						
Not born during Khmer Rouge	81·5 %	81·2 %	0·812	84·2 %	88·0 %	< 0·001
Born during Khmer Rouge	18·5 %	18·8 %		15·8 %	12·0 %	
Child's age (in years)						
Less than 1	7·5 %	29·0 %	< 0·001	7·3 %	25·0 %	< 0·001
1	20·1 %	18·2 %		26·7 %	18·9 %	
2	20·9 %	18·0 %		19·1 %	21·0 %	
3	25·5 %	18·3 %		21·1 %	18·2 %	
4	26·0 %	16·5 %		25·8 %	16·9 %	
Child's sex						
Male	50·4 %	50·7 %	0·805	50·2 %	51·2 %	0·418

Overall, stunted children were from households with lower wealth and lower maternal educational attainment and tended to live in rural areas as compared to non-stunted children in both periods (Table 1). Additionally, in period 2, a greater proportion of stunted children had mothers employed in agriculture, service industries, or manual labour as compared to period 1. Mothers of stunted children tended to be older and had more total births, and a higher fraction were smokers and of short stature than those of non-stunted children. There was also a sizeable reduction in overall income inequality in period 2, such that household wealth appeared more evenly distributed across quintiles in period 2 among both stunted and non-stunted children as compared to period 1. For example, a smaller proportion of the population was classified in the two richest quintiles in period 1 than in period 2 (stunted: period 1–22·2 % *v*. period 2–27·0 %; non-stunted: period 1–34·4 % *v*. period 2–45·6 %).

Descriptive characteristics of the subsample restricted to children of overweight mothers are shown in Table 2. Among this subsample, the proportion of stunted children decreased between periods (36·8 % in period 1 to 23·5 % in period 2, *P* < 0·001). The general pattern of sociodemographic characteristics of these children is quite similar to the overall sample described above. There were no major socio-economic changes over time among households with overweight mothers. But, as among the full sample of children, households restricted to overweight mothers displayed similar wealth trends over time (Fig. 1): The poorest quintile exhibited decreased wealth between periods, while richer quintiles displayed increasingly greater gains in wealth.

**Table 2:** Characteristics of the subsample of children with overweight mothers by period and stunting status (period 1, *n* 459; period 2, *n* 1113)

	Period 1 (2000–2005)			Period 2 (2010–2014)		
	Stunted	Not stunted	*P*-value	Stunted	Not stunted	*P*-value
Total number in group	169	290		262	851	
Household wealth						
Poorest	18·9 %	12·1 %	< 0·001	18·3 %	10·8 %	< 0·001
Poorer	19·5 %	10·0 %		18·7 %	15·0 %	
Middle	14·2 %	15·9 %		19·5 %	16·3 %	
Richer	24·3 %	20·7 %		19·5 %	22·2 %	
Richest	23·1 %	41·4 %		24·0 %	35·6 %	
Maternal short stature						
Not short	62·1 %	79·3 %	< 0·001	60·0 %	74·3 %	< 0·001
Short	37·9 %	20·7 %		40·1 %	25·7 %	
Mother’s highest level of education						
No education	23·7 %	25·2 %	0·026	18·7 %	14·3 %	0·012
Primary education	62·7 %	50·7 %		52·7 %	49·6 %	
Secondary education	13·6 %	23·4 %		27·9 %	31·8 %	
Higher than secondary education	0·0 %	0·7 %		0·8 %	4·2 %	
Maternal employment						
Not working	34·3 %	32·1 %	0·393	25·2 %	33·5 %	< 0·001
Professional/technical/clerical/sales	31·4 %	37·6 %		25·2 %	31·4 %	
Agricultural/services/manual	34·3 %	30·3 %		49·6 %	35·1 %	
Residence						
Urban	23·1 %	34·5 %	0·01	33·6 %	38·4 %	0·157
Rural	76·9 %	65·5 %		66·4 %	61·6 %	
Maternal age						
15–24	7·1 %	13·1 %	0·098	11·1 %	14·2 %	0·240
25–34	48·5 %	49·0 %		63·4 %	57·9 %	
35–49	44·4 %	37·9 %		25·6 %	27·8 %	
Mother’s total number of births						
Mean	4·6	3·71	< 0·001	3·26	2·72	< 0·001
sd	2·63	2·31		1·95	1·56	
Maternal smoking status						
Non-smoker	91·7 %	92·8 %	0·685	93·1 %	97·2 %	0·003
Smoker	8·3 %	7·2 %		6·9 %	2·8 %	
Mother born during Khmer Rouge (1975–1979)						
Not born during Khmer Rouge	87·6 %	90·0 %	0·421	79·8 %	82·4 %	0·340
Born during Khmer Rouge	12·4 %	10·0 %		20·2 %	17·6 %	
Child’s age (in years)						
Less than 1	3·0 %	23·8 %	< 0·001	5·7 %	18·9 %	< 0·001
1	14·2 %	13·4 %		24·0 %	15·9 %	
2	21·9 %	23·4 %		21·8 %	21·5 %	
3	29·0 %	17·9 %		21·0 %	22·1 %	
4	32·0 %	21·4 %		27·5 %	21·6 %	
Child’s sex						
Male	45·0 %	49·3 %	0·369	47·7 %	49·9 %	0·528
Female	55·0 %	50·7 %		52·3 %	50·1 %	

Table 3 displays regression results from four logistic models estimating the associations between SES, maternal and child characteristics with child stunting among the full sample of children in each period. Model 1 included maternal overweight status and wealth quintiles, Model 2 added maternal health indicators and characteristics, Model 3 added child characteristics and Model 4 added residence (urban *v*. rural). Results suggest that maternal overweight status and household wealth had a stronger link with having a stunted child in period 2 than in period 1. For example, in all models, results show that maternal overweight status was not significantly associated with child stunting in period 1 but was protective in period 2 after adjusting for maternal and child characteristics (Model 4, adjusted OR (aOR) = 0·72, 95 % CI: 0·61, 0·85). Children living in the poorest households had higher odds of stunting in period 2 (aOR = 2·61, 95 % CI: 2·073·29) than in period 1 (aOR = 2·10, 95 % CI: 1·69, 2·62). Maternal characteristics were also important in predicting stunting (full models are detailed in Appendix 1): higher number of births, smoking status, and maternal short stature were associated with increased risk of having stunted children in both periods.

**Table 3:** Coefficient estimates from logistic regressions for the association between child stunting and socio-economic, maternal health, and child characteristics among the full sample of children by period (period 1, *n* 7033; period 2, *n* 7955)†

	Model 1: maternal weight status and household wealth‡		Model 2: Model 1 + mother’s characteristics§		Model 3: Model 2 + child characteristics||		Model 4: Model 3 + residence¶	
	aOR	95 % CI	aOR	95 % CI	aOR	95 % CI	aOR	95 % CI
Period 1 (2000–2005)								
Mother overweight (ref, Not overweight)	0·98	0·81, 1·20	0·98	0·80, 1·20	0·95	0·77, 1·18	0·95	0·77, 1·18
Household wealth quintile (ref, richest)								
Poorest	3·01***	2·52, 3·60	1·94***	1·59, 2·38	2·05***	1·66, 2·52	2·10***	1·69, 2·62
Poorer	2·41***	2·01, 2·89	1·73***	1·42, 2·11	1·80***	1·46, 2·21	1·84***	1·48, 2·29
Middle	2·09***	1·73, 2·53	1·62***	1·32, 1·97	1·68***	1·36, 2·06	1·72***	1·38, 2·13
Richer	1·78***	1·46, 2·17	1·51***	1·24, 1·85	1·56***	1·26, 1·92	1·59***	1·28, 1·97
Period 2 (2010–2014)								
Mother overweight (ref, not overweight)	0·79**	0·67, 0·92	0·74***	0·63, 0·87	0·73***	0·62, 0·86	0·72***	0·61, 0·85
Household wealth quintile (ref, richest)								
Poorest	3·31***	2·82, 3·87	2·28***	1·87, 2·77	2·33***	1·90, 2·85	2·61***	2·07, 3·29
Poorer	2·76***	2·34, 3·26	2·09***	1·72, 2·54	2·12***	1·73, 2·59	2·37***	1·88, 2·97
Middle	2·30***	1·93, 2·74	1·88***	1·54, 2·28	1·93***	1·58, 2·35	2·13***	1·71, 2·65
Richer	1·66***	1·39, 1·98	1·50***	1·24, 1·81	1·52***	1·26, 1·85	1·64***	1·33, 2·01

*P < 0·05, **P < 0·01, ***P < 0·001.

† Cluster robust standard errors were used. A full table of results is shown in Appendix 1.

‡ Model 1: child stunting = α + β_1_ maternal weight + β_2_ household wealth + ϵ

§ Model 2 adds the following covariates to Model 1: mother’s education, maternal employment, maternal age, parity, maternal smoking, maternal short stature and mother’s birth during the Khmer Rouge.

|| Model 3 adds the following covariates to Model 2: child’s age and child’s sex.

¶ Model 4 adds residence (urban *v.* rural) to Model 3.

**Fig. 2:**
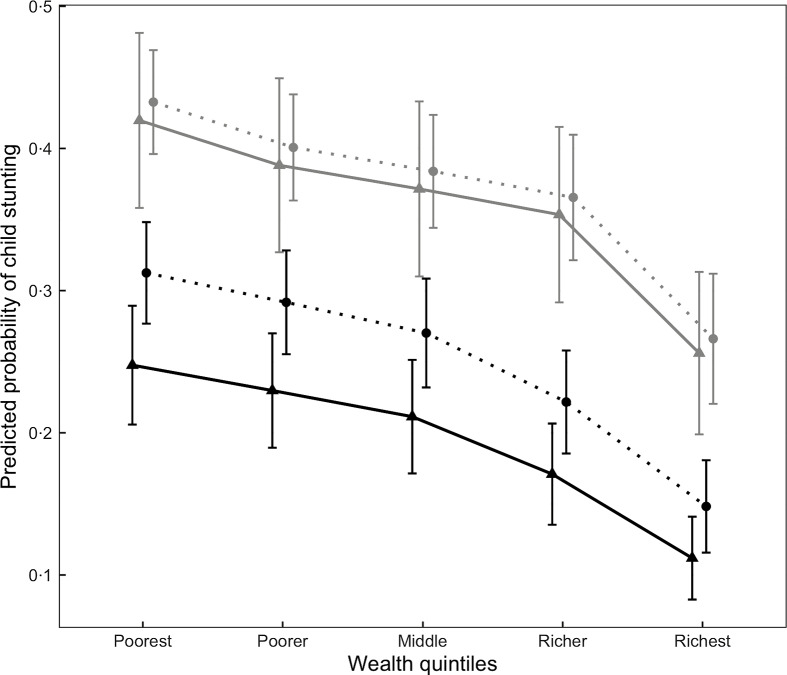
Predicted probabilities of child stunting by wealth quintile, maternal overweight status and period among the full sample (period 1, *n* 7033; period 2, *n* 7955)^1^. 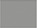, 2000–2005; 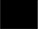, 2010–2014; 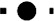, mother not overweight; 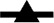, mother overweight. ^1^Predicted probabilities of stunting using coefficient estimates from Table 3, Model 4 among children of mothers aged 30 years, with three total children, non-smokers, not short, with no education, employed in an agricultural, service or manual occupation, rural residence, and not born during the Khmer Rouge famine. Characteristics were selected based on modal values (e.g. smoking status, occupation, etc.) or values close to sample means (e.g. age, number of children). Child’s age and sex were taken at their means. Points were horizontally dodged to show contrast

We plotted predicted probabilities of child stunting by wealth and maternal weight comparing periods 1 and 2 (Fig. 2). Results show that while the adjusted probability of child stunting declined in period 2 relative to period 1 for both overweight and non-overweight mothers across all wealth statuses, there was a larger decline in the probability of child stunting among overweight mothers. For example, children in the poorest households with overweight mothers had about 42 % probability (95 % CI: 36 %, 48 %) of being stunted in period 1 but decreased to about 25 % probability (95 % CI: 21 %, 29 %) in period 2 (a decline of about 17 percentage points) with similar declines in the other wealth quintiles. In contrast, among non-overweight mothers, the probability of child stunting in the poorest households changed from about 43 % in period 1 to about 31 % in period 2, a reduction of about 12 percentage points.

We further studied this result by estimating the association of child stunting among the subsample of children with overweight mothers (Table 4), following a similar modelling approach as with the full sample of children. Results indicate that household wealth continued to be strongly associated with having a stunted child, but maternal characteristics such as employment, short stature and total number of births also played an important role. For example, children of overweight mothers in the poorest household quintile had more than double the risk of being stunted (Model 1, period 1 – aOR = 2·81 95 % CI: 1·58, 5·00; period 2 – aOR = 2·51 95 % CI: 1·60, 3·93) as compared to those in the richest households. After adjusting for additional SES factors, results indicate that maternal education, employment and residence partially mediated the association of wealth and stunting, likely because these factors exacerbated the influence of wealth in period 2 (Model 2, poorest: period 1 – aOR = 2·53 95 % CI: 1·25, 5·13; period 2 – aOR = 2·61 95 % CI: 1·43, 4·77). In particular, mothers working in agricultural, service or manual occupations in period 2 were more likely to have a stunted child than those not working (Model 3, aOR = 1·84, 95 % CI: 1·28, 2·66). In contrast, maternal characteristics, specifically short stature and higher total number of births, were linked with increased likelihood of child stunting in both periods (full models presented in Appendix 2).

**Table 4:** Factors associated with child stunting among the subsample of children with overweight mothers by period (period 1, *n* 459; period 2, *n* 1113)†

	Model 1: household wealth‡		Model 2: Model 1 + SES factors and residence§		Model 3: Model 2 + maternal health indicators||	
	aOR	95 % CI	aOR	95 % CI	aOR	95 % CI
Period 1 (2000–2005)						
Household wealth quintile (ref, richest)						
Poorest	2·81***	1·58, 5·00	2·53*	1·25, 5·13	1·85	0·86, 4·01
Poorer	3·50***	1·88, 6·53	3·16**	1·62, 6·19	2·70**	1·36, 5·37
Middle	1·61	0·87, 2·97	1·46	0·76, 2·80	1·26	0·63, 2·50
Richer	2·10**	1·22, 3·64	1·89*	1·08, 3·32	1·69	0·95, 2·98
Period 2 (2010–2014)						
Household wealth quintile (ref, richest)						
Poorest	2·51***	1·60, 3·93	2·61**	1·43, 4·77	1·96*	1·05, 3·69
Poorer	1·84**	1·19, 2·85	1·92*	1·07, 3·43	1·62	0·88, 2·98
Middle	1·76*	1·14, 2·73	1·88*	1·12, 3·16	1·53	0·89, 2·65
Richer	1·30	0·85, 1·97	1·34	0·85, 2·11	1·25	0·79, 2·00

*P < 0·05, **P < 0·01, ***P < 0·001.

† Cluster robust standard errors were used. A full table of results is shown in Appendix 2.

‡ Model 1: child stunting = α + β_1_ household wealth + ϵ

§ Model 2 adds the following covariates to Model 1: mother’s education, maternal employment and residence (urban *v.* rural)

|| Model 3 adds the following covariates to Model 2: maternal age, parity, maternal smoking, maternal short stature and mother’s birth during the Khmer Rouge.

## Discussion

This study sought to understand trends of simultaneous over- and under-nutrition and how associations with SES – a theoretical driver of the nutritional transition – have shifted over time. Our findings indicate that the characteristics of mothers have changed between periods 1 (2000–2005) and 2 (2010–2014). There was a growing gap in the difference in the probability of child stunting between overweight and non-overweight mothers over time across wealth quintiles, such that maternal overweight status became protective. We observed that maternal overweight status was negatively associated with child stunting in period 2, but not period 1. Two possible mechanisms may explain this finding. First, beginning in the mid-2000s, several relevant national health policies and strategies were implemented, including the National Policy on Infant and Young Child Feeding (2002 and 2008), Health Strategic Plans (2003–2008, 2008–2015) and the National Interim Guidelines for the Management of Acute Malnutrition (2011). These policies targeted maternal and child health and nutrition as a key objective. It is possible that because of improved access to health care and nutrition (e.g. higher quality of food and greater micronutrient intake), child stunting could have improved at a faster pace relative to mothers’ overweight status, and the link between overweight status and child stunting was reversed as a result (hence, the negative association in period 2). This may have resulted in overweight status becoming a protective factor. Second, the implementation of the national health policies did not translate to a lower fraction of overweight mothers; on the contrary, there was a larger fraction of overweight mothers in period 2 than in period 1 for both stunted and non-stunted children. Thus, our result of the negative association in period 2 is likely due to the divergent trend of decreased child stunting and the increased fraction of overweight mothers. Future research may investigate the extent to which Cambodia’s health policies impacted maternal and child well-being.

We found that socio-economic factors, particularly household wealth, have become more important in recent times in relation to the association of mother’s characteristics and stunted children. In addition, our analyses suggest an increasing wealth gap between children in the richest and poorest households: the poorest households appear to have less wealth in the second period, while those in richer quintiles gained wealth. In both periods, analyses indicate that household wealth remains the primary driver of child stunting. In the analysis of stunting in the full sample of children, household wealth remained significant in all models, such that the poorest households had higher probability of child stunting in both periods, indicating strong and independent associations with household wealth.

Overall, the probabilities of stunting decreased over time but estimates also suggest that there are greater differences in the probability of child stunting between the poorest and the richest household wealth quintiles in period 2 as compared to period 1.Children living in the poorest households were more than twice as likely to be stunted than those living in the richest households after controlling for mother’s and child’s characteristics. More importantly, this association is stronger in period 2 than in period 1.This suggests that despite sizeable reductions in overall income inequality (i.e. more even distribution of wealth across the population) in Cambodia over the period of study, the poorest households were more likely to have an increasingly larger share of stunted children.

In our analysis among children of overweight mothers, there was no evidence that wealth inequality changed across periods, in contrast to the pattern of decreasing income inequality observed among the sample of all children. However, we found that factors associated with stunting among this subsample resembled factors associated with stunting in general. Our results also indicated an increasing household wealth gap between richest and poorest, and the association of lower wealth status with stunted offspring remained steady over time. In addition, after adjusting for other SES factors such as maternal education and employment, the odds of child stunting for the poorest compared to richest households were similar over both periods. These results suggest that in spite of overall economic growth, socio-economic factors remained primary factors driving child stunting among households with overweight mothers – the risk for the poorest persisted over time.

This study highlights the influence of socio-economic inequality over time on the double burden of malnutrition in which poor households are at a greater disadvantage than the rich. Though aggregate measures indicate reductions in income inequality over time, our results suggest that the poorest households are left behind and as a result, continue to experience persistently high levels of child stunting. These findings support literature indicating that the phenomenon of households with overweight mothers and stunted children are driven primarily by social and economic inequalities^(46,47)^. Economic development has been cited as a critical driver of the epidemiological transition of under- to over-nutrition^(29)^. However, as economies develop and underlying causes of mortality shift, the poorest continue to experience the worst health^(48)^. Our findings support this pattern: we find that though more mothers were overweight in recent times (a protective factor), the poorest children of overweight mothers still faced high odds of stunting. Several studies regarding the double burden of under and over-nutrition in other countries have also implicated poverty and low SES as key risk factors^(23,46,47,49)^ and observed that this phenomenon is growing at the fastest pace among disadvantaged households^(50)^.

We also found that employment in agricultural, service, and manual occupations became a significant factor for child stunting in period 2. Our findings are consistent with some studies that found that employed mothers were more likely to have stunted children than non-employed women^(51–53)^. However, literature regarding the relationship between maternal employment and child stunting is mixed. One study in Ethiopia found no association between maternal employment and child stunting, and another study in Angola found that maternal employment outside of the house was associated with reduced stunting prevalence^(54,55)^. This suggests that the contextual characteristics of maternal employment, including availability of childcare support, income earned and seasonal unemployment, among others, are critical determinants of food security, feeding practices and dietary diversity^(56–60)^. Further research is needed to understand the relationship between maternal employment in these occupations and child nutrition in Cambodia.

It is notable that associations with household wealth were attenuated after additional adjustment for maternal health characteristics, which suggests partial mediation via maternal health and well-being. Our results show that maternal short stature was particularly salient in both periods, as indicated by more than doubling the odds of stunting. Maternal short stature indicates poor nutritional growth in early childhood^(61,62)^ that may have influenced poor metabolic functioning in adulthood^(4,10,63)^. Studies have also found that maternal short stature is associated with both worse birth outcomes and growth among offspring independent of food and nutrients consumed^(39,64)^. Furthermore, evidence indicates that inadequate childhood growth is linked to persistent disadvantage, including lower educational attainment, reduced adult income and decreased birthweight of offspring^(3,63)^. Our findings support evidence of intergenerational transmission of adversity and underdevelopment and thus underscore the need to intervene in cycles of inequalities^(65–67)^.

This study has some limitations. First, exploration of mediating factors was limited by the data available. We lacked information about critical factors that could help explain the relationship between SES and nutritional status, including dietary information, food insecurity, feeding practices and childcare support. The dataset lacked information about important health factors for a large fraction of children, for example, birthweight or gestational age. Thus, our results may be interpreted conservatively since we were unable to account for these potential confounders in our estimates. The sample size in period 1 was also smaller as compared to the sample size in period 2, which may have contributed to less reliable estimates of association in period 1. Thus, the lack of a negative association between overweight mothers and stunted children in period 1 should be interpreted conservatively. In addition, because we used cross-sectional data, we cannot draw causal conclusions about the associations between maternal weight, SES and child stunting. Moreover, our results should be interpreted with caution as there may be clustering bias introduced by including multiple siblings from the same mothers. Children with siblings in the study differed significantly in age as compared to children with no siblings included (i.e. corresponding to unique mothers) and may have contributed to higher odds of stunting among older children. To mitigate some of these biases, we estimated robust standard errors to correct for clustering by mother.

Finally, Cambodia has made notable social progress in the past few decades, substantially reducing poverty from 47·8 % in 2007 to 13·5 % in 2014^(68)^ and expanding children’s health care in the past several decades, such as increasing child immunisation coverage from 37 % to 93 % of all children^(69)^. However, continued efforts to reduce poverty and additional preventive measures may be needed to address malnutrition and interrupt intergenerational cycles of disadvantage. First, expanding access to early education could be an important strategy to reduce social inequities over a child’s life course. National enrolment in early education for children 3 to 5 years of age was only 41 % in 2016^(70)^. In addition to serving as an important foundation for future educational attainment, early education could serve as a link to nutrition programmes and ensure equitable access to nutritious foods. Because our results also indicate that during 2010–2014 maternal employment was associated with greater odds of child stunting among overweight mothers, access to early education programmes could particularly benefit children of low-income working mothers. Second, while national income inequality has steadily decreased since 2007, efforts are still needed to improve wages and working conditions among certain industries (e.g. agriculture, manufacturing, service) and reduce existing gender wage disparities^(71)^. Formalising employment, ensuring adequate overtime compensation and increasing opportunities for advancement may increase pay, improve working conditions for women and benefit both maternal and child health. Policies that promote equity may measurably reduce relative social disadvantage and mitigate impacts on malnutrition.

In conclusion, we argue that efforts to improve the status and well-being of the poorest households and most disadvantaged groups are needed in order to improve both forms of malnutrition and break intergenerational patterns of physical underdevelopment. Both over- and under-nutrition are posited risk factors for NCD in later life, such as diabetes and CVD, leading causes of death in Cambodia. Furthermore, global trends indicate that the most disadvantaged groups are at greater risk for developing these NCD, yet are least able to bear the costs of needed ongoing treatment^(72,73)^. The problems of over- and under-nutrition should be addressed to reduce health inequities among the poorest groups in society.

## Supplementary material

For supplementary material accompanying this paper visit https://doi.org/10.1017/S1368980021000689. click here to view supplementary material
