# Supplementary material for: Socio-economic status and the double burden of malnutrition in Cambodia between 2000 and 2014: overweight mothers and stunted children
Source: Public Health Nutr. 2021 Feb 26;24(7):1806–17. doi: 10.1017/S1368980021000689 (PMC8094435; doi:10.1017/S1368980021000689)
Supplement: Supplementary file 1 [file S1368980021000689sup.zip › S1368980021000689sup001.pdf]

Appendix 2: All coefficient estimates from logistic regressions for the subsample of children of overweight mothers by period (Period 1, n=459; Period 2, n=1,113)*

	Model 1: Household Wealth		Model 2: + SES factors & Residence		Model 3: + Maternal health indicators	
	aOR	95% CI	aOR	95% CI	aOR	95% CI
**Period 1 (2000-2005)**						
Household Wealth Quintile (ref, Richest)						
Poorest	2.81	(1.58, 5.00)	2.53	(1.25, 5.13)	1.85	(0.86, 4.01)
Poorer	3.50	(1.88, 6.53)	3.16	(1.62, 6.19)	2.70	(1.36, 5.37)
Middle	1.61	(0.87, 2.97)	1.46	(0.76, 2.80)	1.26	(0.63, 2.50)
Richer	2.10	(1.22, 3.64)	1.89	(1.08, 3.32)	1.69	(0.95, 2.98)
Mother's Education (ref, Secondary/Higher)						
No education/Primary	---		0.72	(0.27, 0.41)	0.75	(0.42, 1.32)
Maternal Employment (ref, Not employed)						
Professional/Technical/Clerical/Sales	---		1.08	(066, 1.76)	0.99	(0.60, 1.63)
Agricultural/Service/Manual	---		0.83	(0.52, 1.35)	0.77	(0.47, 1.26)
Rural residence (ref, Urban)	---		1.37	(0.86, 2.16)	1.32	(0.83, 2.11)
Maternal Age	---		---		1.01	(0.97, 1.06)
Total number of births	---		---		1.10	(0.98, 1.23)
Maternal smoking (ref, Non-smoker)	---		---		0.68	(0.32, 1.46)
Maternal short stature (ref, Not short)	---		---		2.15	(1.38, 3.34)
Maternal Birth during Khmer Rouge (ref, not born during Khmer Rouge)	---		---		1.69	(0.91, 3.13)
Constant	0.33	(0.60, 1.40)	0.31	(0.17, 0.55)	0.13	(0.04, 0.45)
**Period 2 (2010-2014)**						
Household Wealth Quintile (ref, Richest)						
Poorest	2.51	(1.60, 3.93)	2.61	(1.43, 4.77)	1.96	(1.05, 3.69)
Poorer	1.84	(1.19, 2.85)	1.92	(1.07, 3.43)	1.62	(0.88, 2.98)
Middle	1.76	(1.14, 2.73)	1.88	(1.12, 3.16)	1.53	(0.89, 2.65)
Richer	1.30	(0.85, 1.97)	1.34	(0.85, 2.11)	1.25	(0.79, 2.00)
Mother's Education (ref, Secondary/Higher)						
No education/Primary	---		0.95	(0.67, 1.34)	1.08	(0.75, 1.55)
Maternal Employment (ref, Not employed)						
Professional/Technical/Clerical/Sales	---		1.33	(0.89, 2.01)	1.42	(0.93, 2.15)
Agricultural/Service/Manual	---		1.75	(1.23, 2.50)	1.84	(1.28, 2.66)
Rural residence (ref, Urban)	---		0.79	(0.54, 1.15)	0.84	(0.56, 1.24)
Maternal Age	---		---		0.97	(0.94, 1.00)
Total number of births	---		---		1.22	(1.08, 1.37)
Maternal smoking (ref, Non-smoker)	---		---		1.43	(0.65, 3.18)
Maternal short stature (ref, Not short)	---		---		1.91	(1.40, 2.59)
Maternal Birth during Khmer Rouge (ref, not born during Khmer Rouge)	---		---		1.21	(0.82, 1.80)
Constant	0.21	(0.16, 0.27)	0.17	(0.11, 0.27)	0.20	(0.08, 0.53)

*Cluster robust standard errors were used
