# Supplementary material for: Socio-economic status and the double burden of malnutrition in Cambodia between 2000 and 2014: overweight mothers and stunted children
Source: Public Health Nutr. 2021 Feb 26;24(7):1806–17. doi: 10.1017/S1368980021000689 (PMC8094435; doi:10.1017/S1368980021000689)
Supplement: Supplementary file 1 [file S1368980021000689sup.zip › S1368980021000689sup002.pdf]

Appendix 1: All coefficient estimates from logistic regressions for the full sample of children by period (Period 1, n=7,033; Period 2, n=7,955)

	Maternal Weight Status & Wealth		Model 2: + Mother's characteristics		Model 3: + Child characteristics		Model 4: + Residence	
	aOR	95% CI	aOR	95% CI	aOR	95% CI	aOR	95% CI
**Period 1 (2000-2005)**								
Maternal weight (ref, Not Overweight)	0.98	(0.81, 1.20)	0.98	(0.80, 1.20)	0.95	(0.77, 1.18)	0.95	(0.77, 1.18)
Household Wealth Quintile (ref, Richest)								
Poorest	3.01	(2.52, 3.60)	1.94	(1.59, 2.38)	2.05	(1.66, 2.52)	2.10	(1.69, 2.62)
Poorer	2.41	(2.01, 2.89)	1.73	(1.42, 2.11)	1.80	(1.46, 2.21)	1.84	(1.48, 2.29)
Middle	2.09	(1.73, 2.53)	1.62	(1.32, 1.97)	1.68	(1.36, 2.06)	1.72	(1.38, 2.13)
Richer	1.78	(1.46, 2.17)	1.51	(1.24, 1.85)	1.56	(1.26, 1.92)	1.59	(1.28, 1.97)
Mother's Education (ref, No education)								
Primary	---		0.83	(0.74, 0.93)	0.80	(0.71, 0.90)	0.80	(0.71, 0.90)
Secondary	---		0.68	(0.57, 0.83)	0.67	(0.56, 0.82)	0.68	(0.56, 0.82)
Higher	---		0.13	(0.02, 0.92)	0.14	(0.02, 0.87)	0.14	(0.02, 0.88)
Maternal Employment (ref, Not [illegible]								
Professional/Technical/Clerical/Sales	---		0.95	(0.80, 1.12)	0.82	(0.69, 0.98)	0.82	(0.69, 0.97)
Agricultural/Service/Manual	---		1.08	(0.97, 1.21)	0.96	(0.85, 1.08)	0.96	(0.85, 1.09)
Maternal Age	---		1.00	(0.99, 1.01)	0.98	(0.97, 0.99)	0.98	(0.97, 0.99)
Total number of births	---		1.06	(1.03, 1.10)	1.08	(1.05, 1.12)	1.08	(1.05, 1.12)
Maternal smoking (ref, Non-smoker)	---		1.29	(1.13, 1.49)	1.33	(1.14, 1.54)	1.33	(1.14, 1.54)
Maternal short stature (ref, Not short)	---		1.65	(1.48, 1.84)	1.81	(1.61, 2.03)	1.81	(1.61, 2.03)
Maternal Birth during Khmer Rouge (ref, not born during Khmer Rouge)	---		1.06	(0.92, 1.22)	0.99	(0.85, 1.14)	0.99	(0.85, 1.14)
Child's Age (ref. <1)								
1 yo	---		---		4.81	(3.98, 5.81)	4.81	(3.98, 5.81)
2 yo	---		---		5.02	(4.17, 6.04)	5.02	(4.17, 6.04)
3 yo	---		---		6.00	(4.99, 7.21)	6.00	(4.99, 7.21)
4 yo	---		---		6.93	(5.75, 8.35)	6.93	(5.75, 8.34)
Child's Sex is Female (ref, Male)	---		---		1.00	(0.90, 1.10)	1.00	(0.90, 1.10)
Rural residence (ref, Urban)	---		---		---		0.94	(0.80, 1.10)
Constant	0.35	(0.30, 0.40)	0.34	(0.24, 0.48)	0.15	(0.10, 0.22)	0.15	(0.10, 0.23)
**Period 2 (2010-2014)**								
Maternal weight (ref, Not Overweight)	0.79	(0.67, 0.92)	0.74	(0.63, 0.87)	0.73	(0.62, 0.86)	0.72	(0.61, 0.85)
Household Wealth Quintile (ref, Richest)								
Poorest	3.31	(2.82, 3.87)	2.28	(1.87, 2.77)	2.33	(1.90, 2.85)	2.61	(2.07, 3.29)
Poorer	2.76	(2.34, 3.26)	2.09	(1.72, 2.54)	2.12	(1.73, 2.59)	2.37	(1.88, 2.97)
Middle	2.30	(1.93, 2.74)	1.88	(1.54, 2.28)	1.93	(1.58, 2.35)	2.13	(1.71, 2.65)
Richer	1.66	(1.39, 1.98)	1.50	(1.24, 1.81)	1.52	(1.26, 1.85)	1.64	(1.33, 2.01)
Mother's Education (ref, No education)								
Primary	---		0.99	(0.87, 1.14)	0.97	(0.84, 1.12)	0.97	(0.84, 1.12)
Secondary	---		0.93	(0.78, 1.11)	0.92	(0.77, 1.10)	0.92	(0.77, 1.10)
Higher	---		0.60	(0.38, 0.95)	0.64	(0.39, 1.02)	0.61	(0.38, 0.99)
Maternal Employment (ref, Not [illegible]								
Professional/Technical/Clerical/Sales	---		1.27	(1.08, 1.50)	1.09	(0.92, 1.29)	1.09	(0.92, 1.29)
Agricultural/Service/Manual	---		1.34	(1.19, 1.50)	1.11	(0.98, 1.26)	1.12	(0.99, 1.27)
Maternal Age	---		0.99	(0.98, 1.00)	0.98	(0.96, 0.99)	0.98	(0.96, 0.99)
Total number of births	---		1.12	(1.07, 1.16)	1.12	(1.08, 1.17)	1.12	(1.07, 1.17)
Maternal smoking (ref, Non-smoker)	---		1.42	(1.17, 1.74)	1.46	(1.19, 1.78)	1.47	(1.20, 1.80)
Maternal short stature (ref, Not short)	---		2.04	(1.83, 2.27)	2.14	(1.92, 2.39)	2.14	(1.92, 2.39)
Maternal Birth during Khmer Rouge (ref, not born during Khmer Rouge)	---		1.17	(1.01, 1.37)	1.17	(1.00, 1.36)	1.16	(0.99, 1.36)
Child's Age (ref. <1)								
1 yo	---		---		5.21	(4.27, 6.35)	5.20	(4.27, 6.34)
2 yo	---		---		3.29	(2.69, 4.04)	3.29	(2.68, 4.03)
3 yo	---		---		4.08	(3.34, 5.00)	4.08	(3.34, 5.00)
4 yo	---		---		5.44	(4.44, 6.67)	5.45	(4.45, 6.68)
Child's Sex is Female (ref, Male)	---		---		1.03	(0.93, 1.14)	1.03	(0.93, 1.14)
Rural residence (ref, Urban)	---		---		---		0.85	(0.72, 1.00)
Constant	0.21	(0.19, 0.24)	0.17	(0.12, 0.24)	0.08	(0.05, 0.11)	0.08	(0.05, 0.12)
